# G protein-coupled receptor 39 activation alleviates oxidized low-density lipoprotein-induced macrophage inflammatory response, lipid accumulation and apoptosis by inducing A20 expression

**DOI:** 10.1080/21655979.2021.1952917

**Published:** 2021-07-21

**Authors:** Lu Chen, Zhengdong Fang, Xiaotian Wang, Xiaojie Sun, Xinbao Ge, Can Cheng, Hejie Hu

**Affiliations:** a Department of Vascular Surgery, Provincial Hospital Affiliated to Anhui Medical University, Hefei City, Anhui Province, China; b Department of Vascular Surgery, The First Affiliated Hospital of USTC, Hefei City, Anhui Province, China

**Keywords:** Atherosclerosis, GPR39, A20, macrophage, inflammatory response

## Abstract

G protein-coupled receptor 39 (GPR39) agonist weakens oxidized low-density lipoprotein (ox-LDL)-induced attachment of monocytes to vascular endothelial cells and thus alleviates atherosclerosis. This study looks at whether GPR39 protects macrophages against ox-LDL-induced inflammation and apoptosis and ameliorates lipid accumulation in atherosclerosis and investigates its mechanism. Following inducement of ox-LDL, the expression of GPR39 and tumor necrosis factor alpha-induced protein 3 (TNFAIP3, also known as A20) in Raw 264.7 cells was detected by RT-qPCR and western blotting. The viability of macrophages treated with GPR39 agonist was detected by a cell counting kit 8 kit. GPR39 and A20 expression in ox-LDL-challenged macrophages was assayed by RT-qPCR and western blot with or without GPR30 agonist. After transfection of small interfering RNA (siRNA)-A20, the expression of pro-inflammatory cytokine tumor necrosis factor-α (TNF-α), interleukin (IL)-1β and IL-6 and anti-inflammatory cytokine IL-10 as well as NF-κB p65 and COX2 was detected. Lipid accumulation was observed through Oil Red O Staining. Total cholesterol (TC) and free cholesterol (FC) in macrophages were detected by commercial kits. Lastly, macrophage apoptosis was observed through TUNEL, and apoptosis-related proteins were detected by western blotting . Results indicated that decreased expression of GPR39 and A20 was observed in ox-LDL-induced macrophages. GPR39 agonist significantly increased A20 expression in ox-LDL-treated macrophages. Furthermore, A20 interference reversed the inhibitory effect of GPR39 agonist on ox-LDL-induced inflammation, lipid accumulation, TC and FC overexpression as well as cell apoptosis. In conclusion, activating GPR39 alleviates ox-LDL-induced macrophage inflammation, lipid accumulation and apoptosis in an A20-dependent manner.

## Graphical abstract

**Figure:**
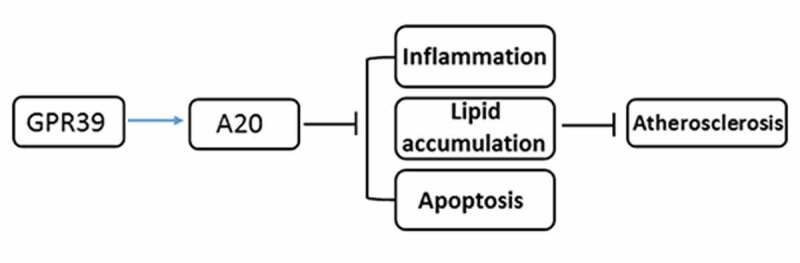

## Introduction

Atherosclerosis is a common cardiovascular disease (CVD) where the elasticity of the artery degrades and the arterial wall hardens over time with local lipid accumulation, smooth muscle cell fibrosis and calcium deposition in the intima of the artery [1–4]. It mainly impacts on large and medium-sized arteries such as the coronary, carotid, cerebral and renal arteries, resulting in oppressive chest pain, angina, myocardial infarction and so on in later life [5,6]. Atherosclerosis is characterized by slow progression, that is to say, the formation of atherosclerosis may start in adolescence and even early childhood [7,8]. Risk factors for atherosclerosis are diverse and complicated, consisting of pathological factors (hypertension, diabetes), genetic factors and lifestyle factors (lack of exercise, smoking, high-fat diet, sleep deprivation, stress) [9–11]. Owing to its considerably high morbidity and poor prognosis, atherosclerosis is a leading cause of heart failure and other CVD-related deaths worldwide [12,13]. The key to the treatment of atherosclerosis lies with early intervention in the risk factors, such as maintaining a healthy lifestyle incorporating routinely physical exercises, low-fat diet and cutting out smoking [14]. However, what makes it difficult is that atherosclerosis can be completely asymptomatic in the early stage of development. And for patients diagnosed with atherosclerosis in a later stage, drug therapy, thrombolysis, stent implantation and coronary artery bypass surgery are the common options, but with unpredictable complications and an overall poor prognosis [15,16].

A recent study pointed out that agonizing the G protein-coupled receptor 39 (GPR39) weakens oxidized low-density lipoprotein (ox-LDL)-induced attachment of monocytes to vascular endothelial cells and thus alleviates atherosclerosis [17]. Another study suggests that GPR39 can augment the secretion of the anti-inflammatory cytokine interleukin (IL)-10 in macrophages to exert an anti-inflammatory effect [18]. These findings inspired our hypothesis that GPR39 may alleviate ox-LDL-induced macrophage inflammatory response and lipid accumulation. Additionally, it has been reported that GPR39 activates the expression of tumor necrosis factor alpha-induced protein 3 (TNFAIP3, also known as A20) and inhibits that of NF-κB inflammatory pathway in vascular calcification [19]. Therefore, we speculated that GPR39 may participate in the regulation of atherosclerosis via an A20-dependent manner.

In the present study, ox-LDL-induced Raw 264.7 macrophage was used to simulate the atherosclerosis model *in vitro*. The expression of GPR39 and A20 was determined. We aimed to investigate that whether GPR39 activation could alleviate ox-LDL-induced macrophage inflammatory response, lipid accumulation and apoptosis as well as the potential regulatory mechanism involved in A20. Our findings might identify a new theoretical basis for targeted therapy for atherosclerosis.

## Materials and methods

### Cell culture and treatment

Raw 264.7 mouse mononuclear macrophages were acquired from Shanghai Cell Bank, Chinese Academy of Sciences and cultured in Dulbecco’s modified Eagle’s medium (DMEM; Hyclone, USA) containing 10% fetal bovine serum (AlphaCell, China) and 1% penicillin/streptomycin in a 37°C, 5% CO_2_ incubator. Ox-LDL (GuideChem, China) in the concentration of 12.5, 25, 50 or 100 μg/ml was chosen to stimulated the macrophages for 12, 24 or 48 h in the presence or absence of 5 or 10 μM of GPR39 agonist TC-G 1008 (ChemGen, China).

### Cell transfection

The small interfering RNA (siRNA) targeting A20 (siRNA-A20-1 and siRNA-A20-2) were constructed by GenePharm (Shanghai, China). The plasmids were transfected into the macrophages using Lipofectamine® 2000 reagent (Invitrogen; Thermo Fisher Scientific, Inc.) in accordance with the manufacturer’s protocol. Transfection efficiency was assessed via reverse transcription-quantitative (RT-q) PCR and western blot analyses.

### Cell counting kit 8 (CCK-8)

The viability of macrophages was detected by a CCK-8 (Beyotime, China) kit. Cells of different treatment groups were harvested and digested with trypsin. Monocyte suspension (5 × 10^4^ cells/mL) was prepared using the culture medium. 100 μL of cell suspension was added to each well of the 96-well plate and incubated with 10 μL of CCK-8 solution for 4 h at 37°C. After oscillation for 10 min, OD_450_ was measured by a microplate reader.

### Oil red O staining

Raw 264.7 cells were inoculated in a 6-well plate. After the culture medium was absorbed, the cells were washed once with phosphate buffer saline (PBS), fixed by 4% paraformaldehyde (Beyotime, China) for 10 min and washed twice with PBS. Next, 1 ml of Oil red O solution (KohyPath, China) was added to each well to stain the cells for 10 min. The cells were finally observed and photographed microscopically.

### Total cholesterol/free cholesterol (TC/FC) quantification

The contents of TC and FC in Raw 264.7 cells were determined using Micro Total Cholestenone (TC) Content Assay Kit and Micro Free Cholestenone (FC) Content Assay Kit (Solarbio, China), following the product instructions.

### Test for the levels of inflammatory factors

The contents of inflammatory factors, including IL-10, IL-1β, IL-6 and tumor necrosis factor-α (TNF-α) in culture medium supernatant were tested with the enzyme-linked immunosorbent assay (ELISA) sandwich method according to the manufacturer’s instructions (Shanghai Xitang Biotechnology Co., Ltd.). The absorbance was measured at a wavelength of 450 nm using a microplate reader (Bio-Rad Laboratories, Inc.).

### Terminal deThe oxynucleotidyl transferase (TdT) dUTP Nick-End Labeling (TUNEL)

For the evaluation of apoptosis, a TUNEL Apoptosis Detection kit (Invitrogen, Carlsbad, Calif, USA) was employed in this study following manufacturer’s recommendations. Raw 264.7 cells were fixed with 4% paraformaldehyde and permeabilized by 0.3% Triton X-100. Cells were then incubated with 50 μl TUNEL reaction buffer. The nuclei were counterstained with DAPI in the dark. Images were visualized and captured using a fluorescence microscope (Olympus Corporation).

### RT-qPCR assay

Total RNA extraction was performed using TRIzol® reagent (Invitrogen; Thermo Fisher Scientific, Inc.). Complementary DNA (cDNA) was obtained through reverse transcription by means of the Prime Script™ RT Master Mix (TaKaRa Bio) in accordance with the manufacturer’s protocol. Subsequently, using cDNA as the template, the gene expression levels were analyzed via qPCR, which was conducted using iTaq™ Universal One-Step iTaq™ Universal SYBR® Green Supermix (Bio-Rad Laboratories, Inc.) on an ABI 7500 instrument (Applied Biosystems; Thermo Fisher Scientific, Inc.). The amplification protocol was 95°C for 30 sec in the holding stage, and 95°C for 5 sec and 60°C for 30 sec in the cycling stage of total 40 cycles. Glyceraldehyde phosphate dehydrogenase gene (GAPDH) were used as internal controls for normalization. The 2^−∆∆Ct^ method was applied to the relative quantification [20].

### Western blot analysis

Cells were collected, lysed by RIPA buffer (BOSTER, China) and centrifuged at 2000 r/min at 4°C for 20 min. Protein concentration was measured in the supernatant by a bicinchoninic acid (BCA) kit (Beyotime, China). Afterward, normalized volumes of samples (40 µg protein per lane) was isolated on sodium dodecyl sulfate-polyacrylamide gel electrophoresis (SDS-PAGE) on a 10% gel and transferred onto polyvinylidene fluoride (PVDF) membranes. The, membranes were blocked with 5% nonfat milk, prior to incubation with primary antibodies for the target proteins. Horseradish peroxidase (HRP)-labeled secondary antibody was then added for incubation for 1 h at room temperature. The immunoreactive protein bands on the membranes were visualized using the Odyssey Infrared Imaging System (LI-COR Biosciences). The intensities of protein bands were quantified by Image J software and the relative protein level was normalized to GAPDH.

### Statistical analysis

The experimental data in this study were analyzed on GraphPad Prism 8.0 (GraphPad Software, Inc., La Jolla, CA, USA). The measurement data are presented as Mean ± Standard Deviation (SD). Contrastive analysis of the measurement data in multiple groups was performed applying one-away analysis of variance (ANOVA) followed by Turkey’s post hoc test, while the date in two groups was compared by Student’s t test. P value less than 0.05 represents statistical significance.

## Results

### Decreased expression of GPR39 and A20 in ox-LDL-challenged macrophages

**Figure 1.**
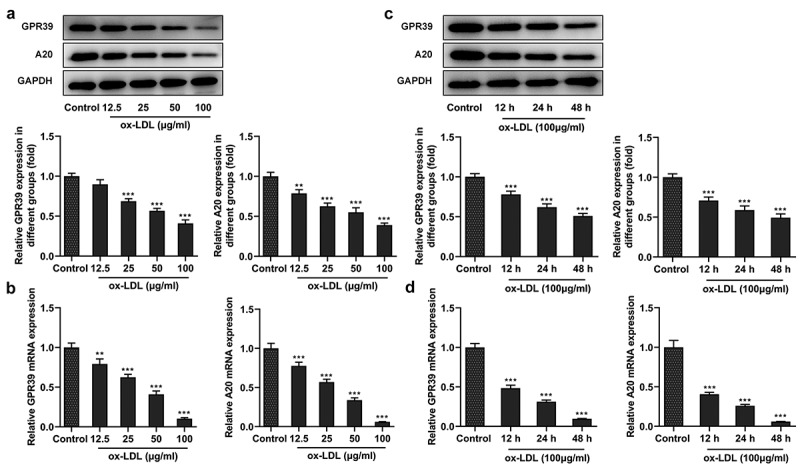
Decreased expression of GPR39 and A20 in ox-LDL-challenged macrophages. (a-b) Relative protein and mRNA expression of GPR39 and A20 after stimulation with 12.5, 25, 50 and 100 μg/ml of ox-LDL, detected by western blot assay and RT-qPCR. (c-d) Relative protein and mRNA expression of GPR39 and A20 after stimulation with 100 μg/ml of ox-LDL, detected by western blot and RT-qPCR assays. **P < 0.01, ***P < 0.001 vs. Control

It has been reported that GPR39 weakens ox-LDL-induced attachment of monocytes to vascular endothelial cells and thus alleviates atherosclerosis [17]. Firstly, the expression of GPR39 was examined in ox-LDL-induced macrophages. Following stimulation with 12.5, 25, 50 or 100 μg/ml of ox-LDL for 48 h, the expression of both GPR39 and A20 in the macrophages showed a reduction with the increase in ox-LDL concentrations compared with the control group (Figure 1(a-b)). 100 μg/ml of ox-LDL was then selected to stimulate the macrophages for 12, 24 and 48 h, after which GPR39 and A20 expression time-dependently decreased (Figure 1(c-d)). Therefore, ox-LDL decreases the expression of GPR39 and A20 in the macrophages.

### GPR39 agonist enhances A20 expression in ox-LDL-challenged macrophages

**Figure 2.**
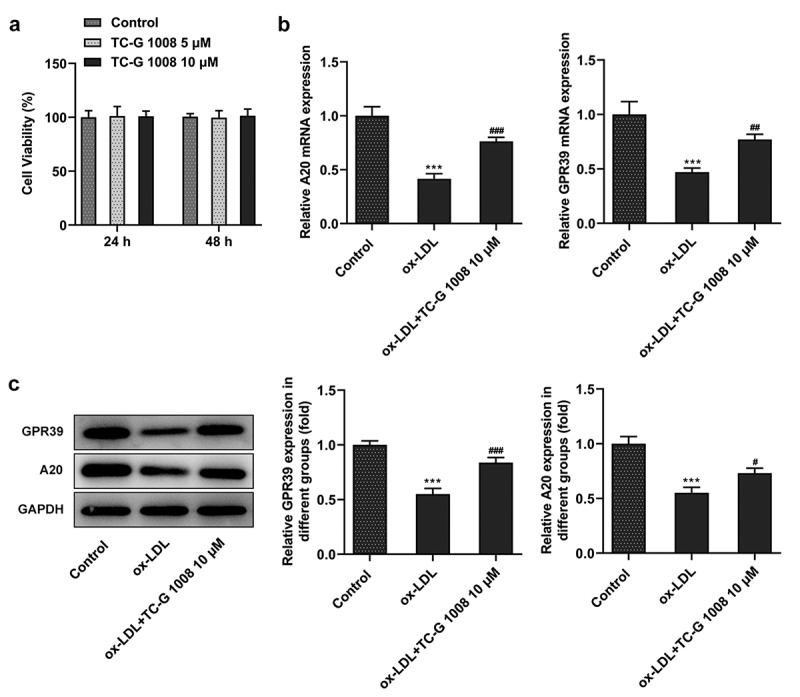
GPR39 agonist enhanced A20 expression in ox-LDL-induced macrophages. (a) The viability of macrophages treated with 5 and 10 μM of TC-G 1008, detected by CCK-8 assay. (b-c) Relative protein and mRNA expression of GPR39 and A20 in ox-LDL-challenged macrophages following treatment with 10 μM of TC-G 1008, detected by western blot analysis and RT-qPCR. ***P < 0.001 vs. Control; #P < 0.05, ##P < 0.01, ###P < 0.001 vs. ox-LDL

Research has proposed that GPR39 can activate the expression of A20 in vascular calcification [19]. Therefore, GPR39 agonist (TC-G 1008) was used to treat Raw 264.7 cells to evaluate the expression of A20. After incubation with both 5 and 10 µM of TC-G 1008 (GPR39 agonist) for 24 and 48 h, the macrophages exhibited no noticeable change in terms of their viability (Figure 2(a)) as comparison to the control group, hence the selection of 10 µM for the following experiments. The expression of both GPR39 and A20 mRNA and proteins in ox-LDL-stimulated macrophages was notably upregulated following treatment with TC-G 1008 when compared with the ox-LDL group (Figure 2(b-c)). These data provide evidence that GPR39 agonist enhances A20 expression in ox-LDL-induced macrophages.

### GPR39 alleviates ox-LDL-induced pro-inflammatory cytokine release in an A20-dependent manner

**Figure 3.**
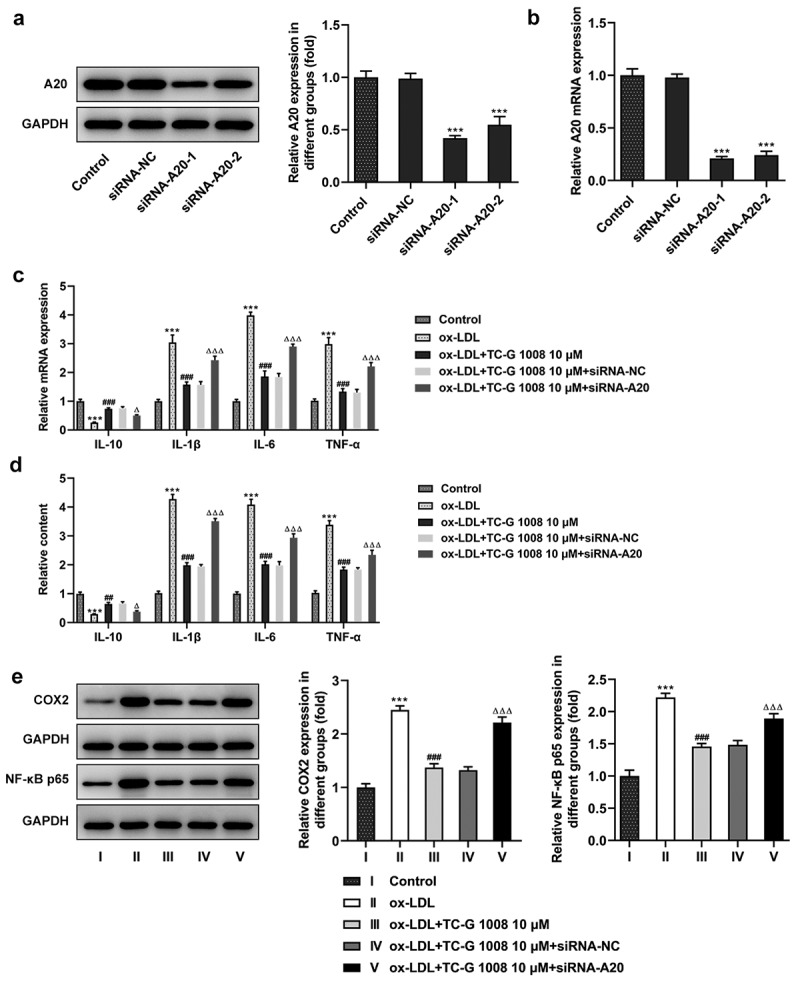
GPR39 alleviated ox-LDL-induced pro-inflammatory cytokine release in Raw 264.7 cells via inducing A20 expression. (a-b) Relative protein and mRNA expression of A20 after transfection of siRNA-A20-1 or siRNA-A20-2, detected by western blotting and RT-qPCR. ***P < 0.001 vs. siRNA-NC. (c-d) Relative levels of IL-10, IL-1β, IL-6 and TNF-α in ox-LDL-challenged macrophages in different treatment groups were detected by RT-qPCR and ELISA kits. (e) Relative protein expression of COX2 and p65 in ox-LDL-challenged macrophages in different treatment groups, detected by western blot assay. ***P < 0.001 vs. Control; ##P < 0.01, ###P < 0.001 vs. ox-LDL; ΔP<0.05, ΔΔΔP<0.001 vs. ox-LDL+TC-G 1008 μM+siRNA-NC

To look at how the interaction between GPR39 and A20 affects ox-LDL-induced inflammatory response of the macrophages, siRNA-A20-1 and siRNA-A20-2) were respectively transfected into the macrophages. The expression of A20 was effectively attenuated afterward compared with the siRNA-NC group, while siRNA-A20-1 had a better knockdown efficacy than siRNA-A20-1 (Figure 3(a-b)), which was thus selected for the subsequent assays. Moreover, the results of RT-qPCR (Figure 3(c)) and ELISA (Figure 3(d)) showed decreased levels of the anti-inflammatory cytokine IL-10 and increased levels of pro-inflammatory IL-1β, IL-6 and TNF-α in Raw 264.7 cells exposed to ox-LDL. TC-G 1008 ameliorated the inflammatory response by enhancing the levels of IL-10 and suppressing that of IL-1β, IL-6 and TNF-α, whereas transfection of siRNA A20 reversed this ameliorative effect. Meanwhile, the protein expression of pro-inflammatory cyclooxygenase 2 (COX2) and NF-κB p65 was also elevated by ox-LDL stimulation as comparison to the control group, which was downregulated in the presence of TC-G 1008 (Figure 3(e)). However, A20 interference again reversed this down-regulatory effect of TC-G 1008 on ox-LDL-induced inflammatory response. These results demonstrate that GPR39 alleviates ox-LDL-induced pro-inflammatory cytokine release in an A20-dependent manner.

### GPR39 improves ox-LDL-induced lipid accumulation in an A20-dependent manner

**Figure 4.**
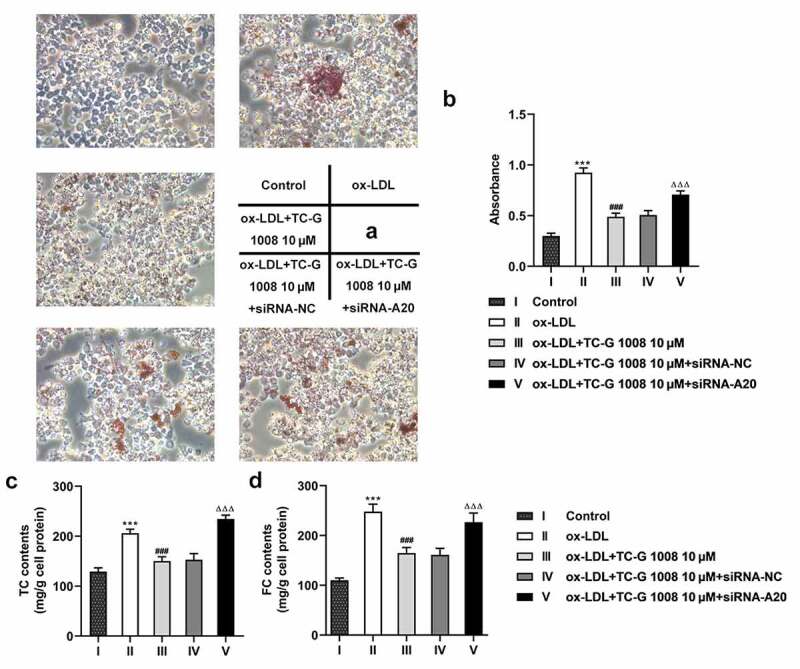
GPR39 improved ox-LDL-induced lipid accumulation of Raw 264.7 cells in an A20-dependent manner. (a-b) Lipid accumulation in ox-LDL-stimulated macrophages in different treatment group, tested with Oil red O staining. (c-d) TC and FC content in ox-LDL-challenged macrophages in different treatment group, determined by commercial kits. ***P < 0.001 vs. Control; ###P < 0.001 vs. ox-LDL; ΔΔΔP<0.001 vs. ox-LDL+TC-G 1008 μM+siRNA-NC

Lipid accumulation is known to be a part of the progress of atherosclerosis [21]. The subsequent experiments were performed to study the effects of GPR39 and A20 in lipid accumulation of macrophages exposed to ox-LDL. It is shown in Figure 4(a-b) that ox-LDL induced accumulating lipid (red) compared with the control group, which was largely ameliorated by treatment with TC-G 1008, whereas A20 interference increased the distribution of the color red compared to the siRNA-NC group. Additionally, a rise in TC and FC content was observed in ox-LDL-challenged macrophages as comparison to the control group, and TC-G 1008 reduced their levels whereas interference with A20 weakened this effect of TC-G 1008 (Figure 4(c-d)). These results indicate the A20-dependent attentive effect of GPR39 on ox-LDL-induced lipid accumulation.

### GPR39 reduces ox-LDL-induced macrophage apoptosis via inducing A20 expression

**Figure 5.**
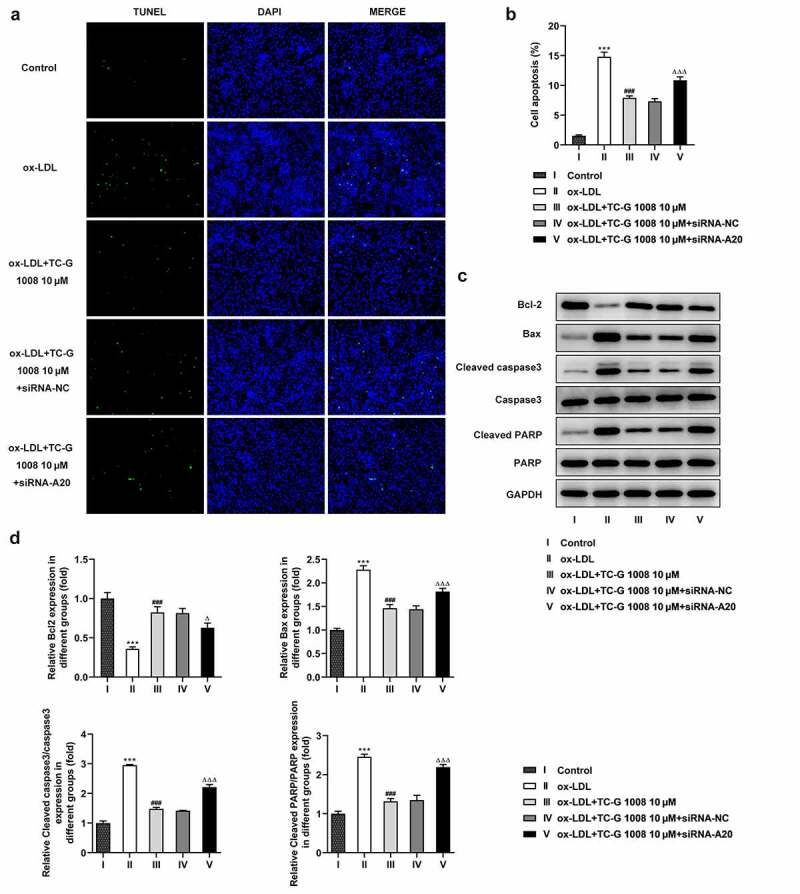
GPR39 reduced ox-LDL-induced macrophage apoptosis via inducing A20 expression. (a-b) The apoptosis of ox-LDL-treated macrophages in different treatment group, measured using TUNEL assay. (c-d) Relative protein expression of apoptosis-related Bcl-2, Bax, cleaved caspase3 and cleaved PARP in macrophages exposed to ox-LDL in different treatment group, detected by western blot assay. ***P < 0.001 vs. Control; ###P < 0.001 vs. ox-LDL; ΔP<0.05, ΔΔΔP<0.001 vs. ox-LDL+TC-G 1008 μM+siRNA-NC

It’s widely accepted that macrophage apoptosis is a process of the formation of necrotic core that acts as an inducer of the long-term low-degree inflammatory stimulation in the intima, which further contributes to the occurrence of atherosclerosis [22]. According to the result of TUNEL assay displayed in Figure 5(a-b), increased number of apoptotic macrophages in the ox-LDL group compared with the control group was notably decreased in the presence of TC-G 1008, which was however rescued after transfection with siRNA-A20. Furthermore, the protein expression of anti-apoptotic Bcl-2 was significantly downregulated and that of pro-apoptotic Bax, cleaved caspase3 and cleaved PARP was conspicuously upregulated in ox-LDL-treated Raw 264.7 cells (Figure 5(c-d)). TC-G 1008 increased Bcl-2 expression and decreased the expression of Bax, cleaved caspase3 and cleaved PARP in Raw 264.7 cells exposed to ox-LDL when compared to the ox-LDL group. However, this effect was abated after transfection of siRNA-A20. Collectively, these results suggest that GPR39 reduces ox-LDL-induced apoptosis of the macrophages in an A20-dependent manner.

## Discussion

Atherosclerosis is the main cause of coronary heart disease, cerebral infarction and peripheral vascular disease around the world [23,24]. Lipid metabolism disorder is the foundation of atherosclerotic lesions [25]. Unfortunately, current treatments are still in need of effectiveness in preventing the development of atherosclerosis and the complications after its onset. In this study, we demonstrated that both GPR39 and A20 expression was remarkably upregulated in ox-LDL-treated Raw 264.7 cells, and GPR39 activation alleviates ox-LDL-induced macrophage inflammatory response, lipid accumulation and apoptosis by inducing A20 expression.

Extensive research has corroborated the association between G protein-coupled receptors (GPCRs) and cardiovascular diseases, including atherosclerosis. For example, Li et al. through their experiments have found that activation of GPCRs contributed to the function of the cardiovascular systems and triggers a series of cellular responses in atherosclerosis [26]. Yu et al. have reported the involvement of G protein-coupled receptor 146 (GPR146) in the regulation of systemic cholesterol metabolism in the development of atherosclerosis [27]. In addition, according to a recent study, strengthening the expression of GPR39 with its agonist TC-G 1008 decreases the attachment of ox-LDL-challenged monocytes to endothelial cells, thus alleviating decelerating the development of atherosclerosis [17]. Another report has mentioned that there exists a positive correlation between the expression of GPR39 and that of A20 and that GPR39 can inhibit the NF-κB signaling pathway in vascular calcification [19]. Extrapolating from the above findings, we presumed that GPR39 may play a role in atherosclerosis through its interaction with A20. Preliminary experiments in our study showed that both the expression of GPR39 and that of A20 decreased significantly in the macrophages following ox-LDL stimulation.

Cellular metabolism of immune cells, such as monocytes and macrophages, is a manipulator of the chronic inflammation in atherosclerosis [28]. GPR39 has proven to augment the secretion of the anti-inflammatory cytokine IL-10 in macrophages to exert an anti-inflammatory effect [18]. Li et al. have found that TNF-α-induced inflammation in human fibroblast-like synoviocytes can be effectively alleviated by the upregulation of GPR39 [29]. Furthermore, A20 has also been shown to mitigate the inflammation alleviate the apoptosis of ox-LDL-stimulated macrophages [30]. Consistent with previous studies, our study illustrated that GPR39 agonist suppressed the release of pro-inflammatory cytokines while improving that of the anti-inflammatory cytokine IL-10 in ox-LDL-challenged macrophages, and that A20 interference reversed the anti-inflammatory effect of GPR39 agonist.

Lipid accumulation is known to be a part of the progress of atherosclerosis. As the lipids gradually accumulate in the arterial wall, macrophages absorb the excessive lipids and eventually grow into foam cells [31]. A high level of cholesterol, an important lipid, in the arterial intima is especially unfavorable for the cardiovascular system and is also a vital biological indicator for atherosclerosis [32]. In a rat model of diabetes, GPR39 was found to play a potential regulatory role in lipid homeostasis, as a high level of its expression was observed in the adipose tissue of obese rats and rats with type 2 diabetes [33]. In our study, GPR39 agonist markedly suppressed lipid accumulation in ox-LDL-challenged macrophages, while this effect was attenuated to a great extent by A20 interference. Likewise, the TC and FC content in ox-LDL-challenged macrophages was reduced by GPR39 agonist, which was increased after A20 knockdown. Additionally, macrophage apoptosis is a process of the formation of necrotic core that acts as an inducer of the long-term low-degree inflammatory stimulation in the intima, which further contributes to the occurrence of atherosclerosis [22]. Inhibiting the apoptosis of macrophages is considered a plausible approach to the treatment of atherosclerosis. Our results showed that GPR39 agonist decreased the number of apoptotic macrophages stimulated with ox-LDL, whereas transfection of siRNA-A20 increased cell apoptosis rate.

## Conclusions

Taken together, findings in this study demonstrated that GPR39 alleviates inflammatory response, functions against lipid accumulation and ameliorates aberrant apoptosis in ox-LDL-challenged macrophages via inducing A20 expression. Data in this study reveal the roles of GPR39 and A20 in atherosclerosis and provides evidence that GPR39 agonist is of great potential in the treatment of atherosclerosis. However, the lack of data about the expression of GPR39 and A20 in the macrophage of patients with atherosclerosis (especially in high ox-LDL patients) is a limitation of the present study, which will be investigated in the next experiments to further support the conclusions in this study.

## Data Availability

The datasets used and/or analyzed during the present study are available from the corresponding author on reasonable request.

## Highlights

1. The expression of GPR39 and A20 is downregulated in ox-LDL-stimulated macrophages.
2. GPR39 agonist enhances A20 expression in macrophages exposed to ox-LDL.
3. GPR39 alleviates ox-LDL-induced inflammation, lipid accumulation and apoptosis.
4. GPR39 improves ox-LDL-induced macrophages injury via inducing A20 expression.
